# Results of an Interdisciplinary Day Care Approach for Chronic Tinnitus Treatment: A Prospective Study Introducing the Jena Interdisciplinary Treatment for Tinnitus

**DOI:** 10.3389/fnagi.2017.00192

**Published:** 2017-06-16

**Authors:** Daniela Ivansic, Christian Dobel, Gerd F. Volk, Daniel Reinhardt, Boris Müller, Ulrich C. Smolenski, Orlando Guntinas-Lichius

**Affiliations:** ^1^Tinnitus-Centre, Department of Otorhinolaryngology, Jena University HospitalJena, Germany; ^2^Institut of Physiotherapy, Jena University HospitalJena, Germany

**Keywords:** chronic tinnitus, treatment, interdisciplinary, day care, prediction, tinnitus questionnaire

## Abstract

**Objective:** Considering the heterogeneity of the symptoms shown by patients suffering from chronic tinnitus, there are surprisingly few interdisciplinary treatments available, and mostly available only for inpatients. In order to provide an interdisciplinary treatment, we developed a day care concept in which each patient was treated by an ENT doctor, a cognitive behavioral therapist, a specialist for medical rehabilitation and an audiologist (Jena Interdisciplinary Treatment for Tinnitus, JITT). The aim of this study was to observe the changes of tinnitus related distress due to interdisciplinary day care treatment and to determine which factors mediate this change.

**Subjects and Methods:** Tinnitus annoyance was measured using the Tinnitus Questionnaire on 308 patients with chronic tinnitus. They were treated in the day care unit over five consecutive days between July 2013 and December 2014. Data were collected before treatment when screened (T0), at the beginning (T1) and at the end of the 5 day treatment (T2), as well as 20 days (T3) and 6 months after treatment (T4).

**Results:** Overall, tinnitus annoyance improved significantly from the screening day to the beginning of treatment, and to a much larger degree from the beginning to the end of treatment. The treatment outcome remained stable 6 months after treatment. Patients with the following symptoms displayed higher tinnitus annoyance at T0: dizziness at tinnitus onset, tinnitus sound could not be masked with background noise, tinnitus worsening during physical stress, comorbid psychiatric diagnosis, higher age and higher hearing loss. Loudness of tinnitus perceived in the right ear correlated with tinnitus annoyance significantly. Demographic, tinnitus and strain variables could only explain 12.8% of the variance of the change in tinnitus annoyance from T0 to T4. Out of 39 predictors, the only significant ones were “sick leave 6 months before treatment” and “tinnitus annoyance at T0.”

**Conclusion:** The newly developed JITT represents a valuable treatment for chronic tinnitus patients with improvement remaining stable for at least 6 months after treatment. Using a large number of variables did not allow predicting treatment outcome which underlines the heterogeneity of tinnitus.

## Introduction

Tinnitus is widely prevalent and is characterized by experiencing ringing, hissing or similar noises in one or both ears without an external acoustic source. This symptom is in its mild form most often transient, but persists in about 5–10% of the population, leading patients to seek treatment (Henry et al., 2005). As tinnitus is a sensory phenomenon, patients usually consult a physician. Moreover, since tinnitus is a symptom that can arise as a consequence of several disorders, it makes the diagnosis and the resulting treatment rather complex. Some of the possible tinnitus causes are exposure to loud noise, presbyacusis, cardiovascular and cerebrovascular diseases, drugs or medication, ear infections/inflammation, head or neck trauma, hyper- and hypo-thyroidism, Menière's disease, otosclerosis, sudden deafness, or vestibular schwannoma (Hoffman and Reed, 2004). The goal within the first 3 months (acute tinnitus) is to find the cause for the symptomatology and to treat it accordingly. However, in almost 50% of cases no physical origin of the tinnitus can be found (Feldmann, 1992; Lenarz, 1992), leaving the medical practitioner without causal treatment options. To make matters worse, the assumed cause of tinnitus is often successfully treated, but without any influence on the tinnitus itself. This argues in favor of a multifactorial cause, which is supported by the high heterogeneity seen in tinnitus patients.

When lasting more than 3 months and no response to medical treatment can be observed, tinnitus is generally considered as a chronic condition. While most people with chronic tinnitus are able to ignore the sound and do not feel impaired by it, approximately 3–5% of the general adult population perceive tinnitus as extremely bothersome, often to such an extent that it is difficult for them to carry out everyday activities (Davis and El Rafaie, 2000). The most prevalent complaints are concentration problems, mood changes as well as problems with sleep and hearing (Tyler and Baker, 1983; Henry et al., 2005). Additionally, high rates of comorbid psychiatric disorders such as depression, anxiety or somatoform disorders are observed in the group of tinnitus patients with bothersome tinnitus (Sullivan et al., 1988; Zöger et al., 2001). Thus, overall the patients are characterized by a rather large heterogeneity of associated symptoms.

One driving question for the development of therapeutic approaches is why some patients suffer from chronic tinnitus and others do not. Auditory aspects such as pitch, loudness and maskability have been found to be insufficient to explain tinnitus distress (Biesinger and Heiden, 1999; Bleich et al., 2001; Hausotter, 2004; Konzag et al., 2006; Hesse and Schaaf, 2007). One theory that tries to explain the co-occurrence of tinnitus and distress is the neurophysiological model of tinnitus (Jastreboff et al., 1996). According to this model, damage to the auditory pathways plays a crucial role in the development of tinnitus, while other parts of the nervous system (e.g. the limbic system) are responsible for developing tinnitus annoyance. Thus, the dysfunctional interplay between the two systems is responsible for the impact of the impairment on everyday life.

Currently, there is no scientifically proven therapy available that can be considered as a cure for chronic tinnitus. According to the American and German tinnitus guidelines (e.g., American Academy of Otolaryngology-Head Neck Surgery, 2014; German Society of Otorhinolaryngology Head, and Neck Surgery, 2015), the only realistic therapeutic goal is the determination of the tinnitus sensitizing antecedents and their therapeutic manageability, as well as the long term habituation to the phantom noise. Taken together, due to the high heterogeneity of tinnitus and the associated symptoms, an interdisciplinary approach for treatment recommends itself. A recent multidisciplinary systematic review emphasizes the combination of tinnitus specific counseling and cognitive behavior therapy. In the case of hearing loss, additional auditory therapeutic measures (e.g. hearing aids or cochlear implants) should be considered. Comorbidities such as depression should be treated additionally and, if necessary, with drugs (Zenner et al., 2016).

Despite the high prevalence of the impairment, there are only few specialized treatment centers in Germany, and the few existing recommendations for treatment are only rarely fulfilled in clinical practice (Hoare et al., 2012). Very often only inpatients receive interdisciplinary treatments, which is a financially expensive approach. To avoid high expenses, we implemented an interdisciplinary tinnitus treatment in our day care unit. The goal of the treatment was to reduce tinnitus annoyance by addressing the most frequent symptoms that patients with chronic tinnitus complain about: fear of tinnitus, problems with sleep and hearing, inability to relax and concentration problems. The individual treatment was tailored to the specific needs of a patient to account for the individual occurrence and combination of symptoms. Accordingly, we call this approach Jena Interdisciplinary Treatment for Tinnitus (JITT).

The aim of this study was (a) to observe the changes of tinnitus-related distress due to JITT, (b) to investigate in which patients tinnitus annoyance was most strongly expressed at the beginning of the treatment and (c) to explore if treatment success can be predicted.

## Materials and methods

### Assessment of patients

The study was conducted in the Tinnitus-Center at the ENT department of Jena University Hospital, including one screening day, 5 days of day care treatment as well as two follow-up examinations (20 days and 6 months after treatment).

On the screening day all tinnitus patients underwent an examination by an ENT doctor, including ear microscopy, tinnitus case history and history of other ENT symptoms (particularly dizziness). All patients received routine audiometric evaluation including discrete-tone threshold testing and speech audiometry. Audiometry and tinnitus matching were done with calibrated audiometer (MAICO KS5) over Telephonics (TDH 39) headphones. Hearing level (HL in dB) was determined at following frequencies: 125, 250, 500, 750, 1,000, 1,500, 2,000, 3,000, 4,000, 6,000, and 8,000 Hz for the right and left ear for each individual. Pure tone average thresholds (4 PTA) were calculated over the frequencies of 500, 1,000, 2,000, and 4,000 Hz according to the WHO-standard (International Bureau for Audiophonology, 2017). In a second step, the frequency/pitch of the tinnitus was determined. When the patient reported binaural tinnitus, pitch was matched for each ear individually. In case of multiple tinnitus it was suggested that one should concentrate on the most troublesome tinnitus. The patients were asked whether the tinnitus sounds like a pure tone as just perceived during the audiometry, or if it sounds like a broad band or a narrow band noise. Under this directive pure tones or narrow bands of noise or broad band noise were presented to the tinnitus ear. If the pure tone threshold was too high to perceive a test signal at the side of the tinnitus, the contralateral better ear was used to present the sound. When tinnitus pitch was determined, subject's threshold was determined at that frequency. The procedure started at the frequency determined during pitch matching and at a level just below threshold. Then the intensity was increased in 1-dB steps until the patient signaled a match.

In cases of dizziness, vestibular testing was conducted. If indicated (e.g., asymmetric hearing loss, vertigo, headache), other diagnostic procedures (e.g., magnetic resonance imaging) or drug treatment were performed prior to treatment.

Tinnitus-related distress was assessed with the Tinnitus Questionnaire (TQ; Goebel and Hiller, 1998), a standard measure to differentiate patients with mild and severe tinnitus distress based on Hallam's Tinnitus-Questionnaire (Hallam, 1996). Specific fields of distress were assessed by subscales labeled as emotional and cognitive distress, intrusiveness, hearing problems, sleep disturbances, and somatic complaints evoked by the tinnitus. A total sum score ranging 0–30 implies mild tinnitus annoyance (grade 1), 31–46 moderate tinnitus annoyance (grade 2), 47–59 severe tinnitus annoyance (grade 3) and 60–84 very severe tinnitus annoyance (grade 4). The TQ is a standard questionnaire in tinnitus research showing good reliability in terms of retest-reliability (*r* = 0.94) and internal consistency (Cronbachs α = 0.94). Validity coefficients are moderate to high for psychological distress (*r* = 0.5 up to >0.7) and high for tinnitus annoyance (*r* = 0.69–0.74). There is no agreement regarding which treatment-related change in the TQ-score is needed in order for a tinnitus condition to be considered as “improved” (Hall, 2016). The given relevant improvement of the score ranges from an absolute reduction of 5 points to a relative 20% reduction in TQ (Hiller and Haerkötter, 2005; Langguth et al., 2014).

Tinnitus is considered to be at a decompensated level (permanent annoyance and psychological strain) with a TQ score of 47 points or higher (grade 3 and 4) and to be at a compensated level (low secondary symptoms) at 46 points or lower (grade 1 and 2) (Lenarz, 1992; Goebel and Hiller, 1998; Stobik et al., 2005; Mazurek et al., 2009). Therefore, we defined a clinically relevant change as a change from a decompensated to a compensated level, i.e., below 47 points.

Screening of psychological symptoms included a semi-structured interview with a clinical psychologist as well as the full German version of the Patient Health Questionnaire (PHQ; Spitzer et al., 1999), consisting of somatic symptom, depressive mood, anxiety and stress scales. Patients with acute suicidal tendencies or severe psychiatric diagnoses, which prevented a benefit from the day care program, were referred to other specialists.

**Figure 1:**
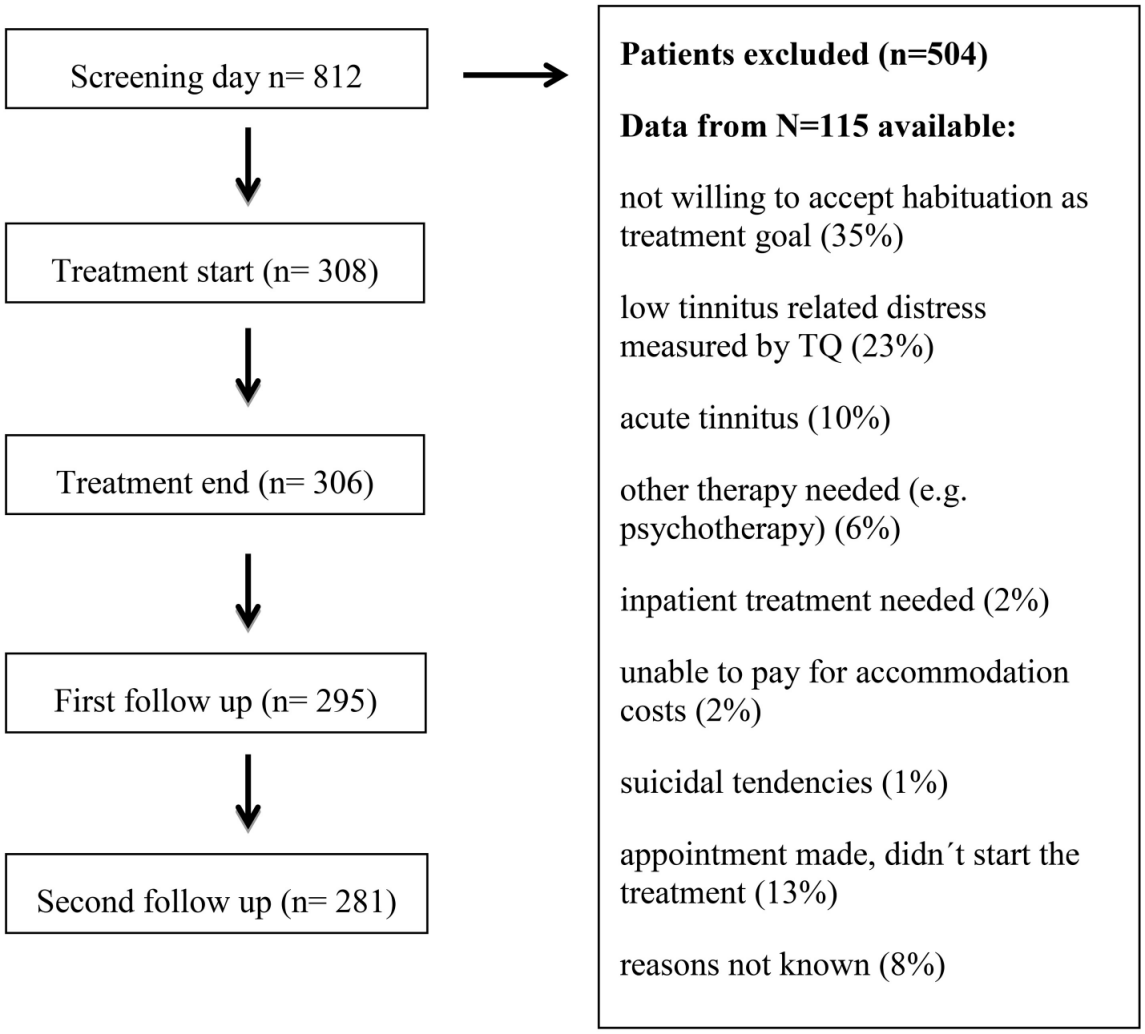
Patient flow chart.

For the treatment to be covered by health insurance, two main inclusion criteria were mandatory: moderate to very severe tinnitus-related distress (measured by TQ) and tinnitus duration of more than 3 months (chronic tinnitus). Tinnitus patients who fulfilled these inclusion criteria and who accepted the treatment goal of tinnitus habituation were included. On average, one out of three tinnitus patients with an appointment in the ENT outpatient department fulfilled the inclusion criteria for the day care treatment. From those not included in the treatment, only 115 patients provided informed consent that the gathered data can be analyzed for scientific purposes. These data are presented in Table 1. The most frequent reasons for exclusion in this group were unwillingness to accept habituation as treatment goal (35%), low tinnitus-related distress measured by TQ (23%) or acute tinnitus (10%). If symptoms of anxiety or depression appeared too severe for treatment participation, patients were transferred to the psychiatric or psychotherapeutic department. Patient flow is shown in Figure 1.

**Table 1:** Baseline characteristics of tinnitus sample (*N* = 308) and excluded tinnitus-patients (*N* = 115).

**Parameter**		**Experimental Group (*N* = 308)**		**Excluded (*N* = 115)**		
Gender: male		52%		50%		
Age (M ± sd)		57.08 ± 12.05		58.53 ± 16.55		
Tinnitus duration (M ± sd)		85.22 ± 94.88		104.95 ± 120.77		
Tinnitus-annoyance (TQ)		52.39 ± 11.92		48.68 ± 14.17		
Tinnitus grade	2 (mild)	32.8%		46.4%		
	3 (severe)	39.9%		28.2%		
	4 (very severe)	27.3%		22.7%		
Tinnitus duration	<1 year	16.6%		20.0%		
	1–5 years	37.3%		27.3%		
	>5 years	46.1%		52.7%		
Number of tinnitus sounds	1	74.7%		–		
	2	21.4%		–		
	3	1.9%		–		
Hyperacusis		57.1%		–		
Comorbid psychiatric disorder		20.5%		10%		
		**Ear:**		**Ear:**		
**Parameter**		**Left**	**Right**	**Left**	**Right**	
Hearing loss in dB (4 PTA: M ± sd)		28.58 ± 18.79	29.63 ± 21.22	33.27 ± 19.29	31.28 ± 16.57	
**Parameter**	**Left**	**Right**	**Both**	**Left**	**Right**	**Both**
Tinnitus localization	20.5%	15.9%	63.6%	15.2%	14.3%	70.5%
Hearing aid at baseline	5.3%	3.9%	41.9%			
Sound generator at baseline	0.3%	0.3%	0.3%			
Cochlear implant at baseline	0	0.3%	0			

*4 PTA, pure tone average for 500 Hz, 1, 2, and 4 kHz; dB, decibel; M, arithmetic mean; sd, standard deviation*.

### Patient sample and treatment

Participants were 308 patients with chronic tinnitus, who fulfilled the above inclusion criteria and were treated in the day care unit of the ENT department of Jena University Hospital between July 2013 and December 2014 (the Tinnitus Center was founded in July 2013 and December 2014 served as the deadline for patient inclusion in the current study). Fifty-two Percent were male participants. The mean age of the sample was 57.08 years (±12.05, ranging from 22 to 81 years). Tinnitus onset was approximately 7 years (85.22 ± 94.88 months, ranging from 3 to 602 months) before treatment. At the first appointment, the tinnitus annoyance indexed by the TQ was 52.39 ± 11.92 points, which is considered as severe. Baseline characteristics of the patient population can be found in Table 1.

The interdisciplinary day care treatment lasted for 5 days (Monday to Friday) with an average of 7 h of therapy per day. Group therapy has been shown to be as effective as individual therapy (universality of the symptom, interpersonal learning, imparting of information, direct advice, imitative behavior, and instillation of hope for more see Yalom, 1995; Andersson and Lyttkens, 1999; Olderog, 1999). For this reason, about 80% (23 h/Week) of our day care treatment was conducted in closed groups of four to six tinnitus patients. Apart from the group therapy, every patient received several individual therapy sessions (6–7 h/Week) and was treated by an ENT doctor, a cognitive behavioral therapist, a specialist for medical rehabilitation and an audiologist. Therefore, the treatment was conducted within 4 modules, which will be described below. The selection of the modules is based on the “Algorithm for the Diagnostic & Therapeutic Management of Tinnitus” (Tinnitus Research Initiative: Biesinger et al., 2008) and the German S3 guideline for chronic tinnitus, in accordance with the recommendations from Zenner et al. (2015).

### Module 1: ENT counseling

Tinnitus-specific counseling has been repeatedly proven to be an effective way of diminishing tinnitus-related distress (Coles, 1995; Henry and Wilson, 1996; Hall and Ruth, 1999; Mazurek et al., 2006). For this reason, ENT doctors conducted tinnitus-specific counseling in a group setting according to the neurophysiological model of tinnitus (Jastreboff et al., 1996). The anatomy of the ear and auditory system, hearing processes as well as hearing impairment and possible mechanisms of tinnitus generation were explained. The benefit of sound therapy with sound enrichment, masker and hearing aids were discussed and patients' questions were answered. ENT counseling was performed in 3 h in group settings. In the individual sessions with ENT doctors, the tinnitus case history was taken into account and all diagnostic outcomes were explained in detail, setting a basis for an individual tinnitus model from a somatic point of view. Individual treatment options for the time after tinnitus day care treatment were discussed and planed.

### Module 2: cognitive behavioral therapy (CBT)

Considering the problems reported as tinnitus related (concentration loss, sleeping disorder, inability to relax, anxiety, depression, fear of aggravation, etc.), it is no surprise that CBT is one of the most validated treatments that reduces tinnitus-related distress (Frenzel, 1998; Andersson and Lyttkens, 1999; Olderog et al., 2004; Martines-Devesa et al., 2010; Cima et al., 2012; for a systematic review see Hesser et al., 2011b). There is a series of studies evidencing the effectiveness of CBT as an internet-based version (Andersson et al., 2002; Kaldo et al., 2008, 2013; Abbott et al., 2009; Hesser et al., 2011b, 2012; Jasper et al., 2014; Weise et al., 2016).

CBT was based on Delb et al. (2002) and Ivansic-Blau (2012) and was administered in 8 group sessions in closed group over 4 days. At first, the roles of attention and emotion in hearing process were explored. The vicious circle of tinnitus distress was explained and factors increasing/decreasing tinnitus awareness were explored. The habitation model of tinnitus (Hallam et al., 1984) was presented, which states that every unknown sound induces an orientation reaction of our body and increases attention to it. If the signal does not change, appears repeatedly and is not considered important, the amount of attention to the signal will reduce as a consequence of habituation. Most tinnitus patients habituate well to their tinnitus sound. Beliefs and emotions hindering habituation were analyzed. The ABC model from Rational-emotive therapy (RET; Ellis, 1993) was introduced and adapted to tinnitus. According to this model, people's beliefs (B) about one activating event (A) strongly affect their emotional and behavioral functioning (C), and not the event itself. Attention switching techniques were learned. Patients were educated about acute/chronic stress responses and about stress reduction techniques. Many tinnitus patients suffering from sleep problems consume alcohol or some medication to improve sleep. We taught our patients about normal sleep patterns and discussed how tinnitus influences sleep. Sleep hygiene recommendations were given and a beneficial sleeping environment (e.g., sound enriched) for tinnitus patients was explored.

In the individual therapy sessions with the CBT therapist an individual tinnitus model was developed, taking into account the following questions: Which individual factors accounted for the tinnitus onset? Which factors helped in dealing with tinnitus? What prevented the implementation of positive management strategies in everyday life? If necessary, patients were encouraged to apply for outpatient psychotherapy subsequent to the treatment in the Tinnitus-Center.

### Module 3: physiotherapy

One subtype of tinnitus is related to a dysfunction of the cervical spine, called cervicogenic somatic tinnitus. Previous research has shown that cervicogenic somatic tinnitus is present in 36–43% of the overall tinnitus population (Abel and Levine, 2004; Fabijanska et al., 2014; Ostermann et al., 2014; Michiels et al., 2015). For that reason, all tinnitus patients underwent physical examination by specialists for medical rehabilitation. In addition to a routine checkup, the influence of head movement, chewing or posture on tinnitus was examined. Individual therapy options for the time after the treatment were discussed and trained. Group physiotherapy was administrated on three consecutive days in 9 sessions, in duration of 50 min each. A physiotherapist conducted one session progressive muscle relaxation (Jacobson, 2006), one session back therapy training and one session physical therapy every day. The goal of the physiotherapy module was to teach patients stress reduction techniques on the one hand, and to expose the patients to ameliorating movement patterns on the other hand. As dysfunctional movements are a common cause for tinnitus aggravation, many tinnitus patients avoid exercise, losing in turn methods for stress reduction.

### Module 4: hearing therapy

**Figure 2:**
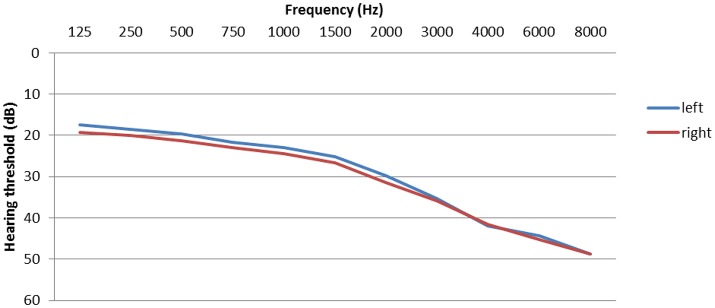
Hearing thresholds.

Hearing problems are one of most prevalent complaints of tinnitus patients. Significant hearing loss is found in 70–80% of tinnitus patients (Henry et al., 2005; Hesse and Schaaf, 2015) and the tinnitus frequency is usually within the range of the greatest hearing loss (Noreña et al., 2002). Hearing levels are displayed in Figure 2. However, inner ear damage is not necessarily obvious in the pure tone audiogram (Weisz et al., 2006; Schaette and McAlpine, 2011; Epp et al., 2012; Tan et al., 2013). Tinnitus patients with normal hearing thresholds have also more difficulties to understand speech in situations with background noise than persons without tinnitus (Hennig et al., 2011). Increased excitation, plasticity, and connectivity along the entire central auditory path can be compensatory responses to the reduced sensory input (De Ridder et al., 2011; Galazyuk et al., 2012; Stein et al., 2013).

To increase the sensory input, at the beginning of day care treatment, every patient was binaurally fitted with hearing aids and received Terzo® hearing therapy (Funk et al., 2008). Terzo® hearing therapy was originally developed for patients with profound hearing loss and combines hearing aid fitting with auditory speech-in-noise training (Terzo® auditory training). In this 1 h per day training, different stimuli and tasks (e.g., a sentence) were administered from a CD player and the responses (e.g., so-called “key words”) were written down in a workbook. Similar to everyday life listening situations, tasks were presented with competing background noise. The Terzo® auditory training was available in three different difficulty levels defined by the initial signal-to-noise ratio (SNR). Depending on listening and comprehension abilities without hearing aids, the suitable training was individually selected. Signal-to-noise-ratio was adaptive and reduced every 2 days in 2 dB SNR. A key section of the workbook contained answers to all exercises, giving the tinnitus patients an important feedback of what was said in the particular sentences. This should increase the awareness for personal hearing (dis)ability. The hearing therapy lasted for 25 days starting at the first day of day care treatment and was continued at home. Terzo® hearing therapy is currently under evaluation; the results will be presented elsewhere.

### Follow-up

The first follow-up measurement took part 20 days after the end of the day care treatment. All patients completed the TQ and gave anonymous feedback about the treatment, involving ratings of each treatment module as “very helpful,” “helpful,” “somewhat helpful,” and “not helpful.” Hearing aid log data were uploaded and Terzo® hearing therapy workbooks were evaluated. In an individual session with an ENT doctor, it was considered whether patients wanted to continue wearing hearing aids in the future or not. If so, hearing aids were subscribed.

The second follow-up was conducted 6 months after the end of day care treatment. In the individual session with an ENT doctor the treatment was evaluated once more, and patients had a possibility to ask questions or inquire about further help. In a group session with a CBT therapist the personally most useful strategies for reduction of tinnitus-related distress were summarized and problems with implementation of the new, favorable behaviors in everyday life were discussed. All patients completed the TQ once more.

Data were collected before the treatment when each patient was screened (T0), at the beginning (T1) and at the end of the 5 day treatment (T2), as well as 20 days (T3) and 6 months after treatment (T4). In case of missing data (Figure 1), we used the last observation carried forward method (Bortz and Döring, 2006). The dependent variable was tinnitus annoyance measured with TQ and its subscales (emotional and cognitive distress, intrusiveness, hearing problems, sleep disturbance, and somatic complains). To identify differences over time, repeated measures analysis of variance (ANOVA) was performed. When significant, *post hoc* testing with Bonferroni correction was performed. To determine the magnitude of change between two points of assessment, effect sizes (ES) using the *d* statistic of Cohen (1992) were calculated; ES between 0.2 and 0.5 are considered as small, from 0.5 to 0.8 as medium and higher than 0.8 as large. The possible impact of patient characteristics on tinnitus annoyance at T0 was tested with Wilcoxon rank sum tests for dichotomized values or with correlations for variables with an interval scale. Regression analysis was used to investigate factors associated with a change in tinnitus annoyance due to treatment. The dependent variable in the regression analysis was change in TQ scores between T0 and T4. The following variables were included in the first regression analysis as independent variables:

- Demographics: age, gender
- Tinnitus-related: duration, localization, frequency, type of onset, subjective loudness, maskability, type of sound, number of sounds, presence during the day, change due to somatic factors like head or jaw movements, change to psychological factors like stress
- Strain variables: baseline TQ score, hearing loss, sound intolerance, sick leave 6 months before treatment, somatic symptoms, depressive mood, anxiety, stress, comorbid psychological disorder
- Otological comorbidity: Menière's disease, dizziness, ear barotrauma, sudden hearing loss, otosclerosis, chronic otitis media, vestibular schwannoma, acoustic trauma

In a second regression analysis, we additionally included “early change” as a predictor. This variable was defined as the TQ change from T0 to T1, i.e., before the actual day care treatment began.

This study was carried out in accordance with the recommendations of ICH harmonized tripartite guideline for Good Clinical Practice, as well as the Declaration of Helsinki. All 308 study participants gave their written informed consent that they were willing to take part in the treatment and that the gathered data can be analyzed for scientific purposes. The protocol was approved by the ethics committee of the Jena University Hospital (4366-03/15).

## Results

### Treatment compliance

Overall, treatment compliance was excellent with very low dropout rates (0.6%, *N* = 2) and with 95.2% of patients taking part in all sessions. According to data logs from the hearing aids at the first follow-up, hearing aids were used for 7.80 ± 4.13 h/day for 21.64 ± 7.79 days (87% of intended 25 days). Almost 90% (89.86%) of the patients completed all tasks of the hearing training.

The anonymous questioner ratings of treatment modules at the end of the treatment showed that all modules were mostly rated as “very helpful” or “helpful”: ENT counseling 99%, CBT 99%, physiotherapy 92%, and hearing therapy 92%. As an example, 99% of patients indicated that they would recommend the treatment to family members if they would suffer from chronic tinnitus.

### General treatment effects

Our treatment goal was to reduce the tinnitus-related annoyance. The repeated measures ANOVA showed that there was a statistical significant difference in tinnitus annoyance measured with TQ over time (*F*_(4, 304)_ = 202.201, *p* < 0.001).

The *post hoc* analysis for tinnitus distress showed a significant reduction from T0 to T1 (*t*_(307)_ = 6.737, *p* < 0.001; from 52.36 ± 11.95 to 48.79 ± 13.74; Cohen's *d* = 0.29, i.e., a small effect), and an even larger reduction from T1 to T2 (*t*_(307)_ = 22.710, *p* < 0.001; from 48.79 ± 13.74 to 34.29 ± 14.98; Cohen's *d* = 1.51, i.e., a large effect). In comparison to T2, significant changes in tinnitus distress were observed neither at T3 (*t*_(307)_ = 0.021, *p* = 0.983), nor at T4 (*t*_(307)_ = 0.378, *p* = 0.706), implying that the outcome remained stable for at least 6 months.

To investigate in more detail which specific tinnitus-related problems did undergo changes over time, we performed repeated measures ANOVA for each TQ subscale. The results are summarized in Table 2. The overall changes over time in all TQ subscales were significant (*p* < 0.001). The *post hoc* analyses showed that the results in 4 subscales (emotional and cognitive distress, intrusiveness and sleep disturbance) were significantly reduced from T0 to T1 and from T2 to T3, but remained stable from T2 to both T3 and T4. In two subscales, namely in subscales “hearing problems” and “somatic complains,” no change appeared from T0 to T1, but there was a significant reduction from treatment begin (T1) to treatment end (T2), remaining stable at T3 and T4.

**Table 2:** Tinnitus Questionnaire subscale scores over time and results of repeated measures analysis of variance (ANOVA).

**TQ subscales (Min-Max)**	**T0 Baseline M (sd)**	**T1 Therapy begin M (sd)**	**T2 Therapy end M (sd)**	**T3 1st follow up (2.5 weeks) M (sd)**	**T4 2nd follow up (6 months) M (sd)**	**F**	**df**	p^*^
Hearing problems (0–14)	7.21 (3.59)	7.36 (3.37)	5.57^a^^,^^b^ (3.22)	5.49^a^^,^^b^ (3.37)	5.40^a^^,^^b^ (3.42)	53.18	4/304	<**0.001**
Emotional distress (0–24)	14.77 (4.31)	13.23^a^ (4.82)	8.84^a^^,^^b^ (4.88)	8.74^a^^,^^b^ (5.26)	8.57^a^^,^^b^ (5.55)	189.37	4/304	**<0.001**
Cognitive distress (0–16)	10.15 (3.28)	8.94^a^ (3.71)	5.41^a^^,^^b^ (3.82)	5.67^a^^,^^b^ (4.02)	5.84^a^^,^^b^ (4.18)	184.20	4/304	**<0.001**
Intrusiveness (0–16)	12.62 (2.22)	12.05^a^ (2.61)	9.07^a^^,^^b^ (3.22)	9.08^a^^,^^b^ (3.60)	9.18^a^^,^^b^ (3.79)	133.97	4/304	**<0.001**
Sleep disturbance (0–8)	4.67 (2.36)	4.41^a^ (2.42)	3.30^a^^,^^b^ (2.54)	3.21^a^^,^^b^ (2.59)	3.11^a^^,^^b^ (2.56)	68.16	4/304	**<0.001**
Somatic complains (0–6)	2.91 (1.85)	2.81 (1.91)	2.10^a^^,^^b^ (1.76)	2.09^a^^,^^b^ (1.88)	1.93^a^^,^^b^ (1.80)	39.02	4/304	**<0.001**

TQ, Tinnitus Questionnaire (Goebel and Hiller, 1998); M, arithmetic mean; sd, standard deviation; Min–Max, Range of subscale;

a Significant change (p < 0.05 corrected) to baseline according to paired t-test with Bonferroni correction;

b Significant change (p < 0.05 corrected) to T1 according to paired t-test with Bonferroni correction;

* *Significant values (p < 0.05) in bold*.

### Predictors of tinnitus annoyance at baseline

To identify which patient subgroups suffer more from tinnitus, we dichotomized the tinnitus patients in subgroups according to baseline characteristics (e.g., tinnitus onset involving/not involving pressure in ears), and compared differences in tinnitus annoyance between these subgroups at T0. If variables were continuous (e.g., tinnitus duration), we correlated them with TQ sum scores at T0. The results are presented in Tables 3, 4.

**Table 3:** Baseline difference in tinnitus annoyance measured with Tinnitus Questionnaire between patient-subgroups.

**Variable**	**Tinnitus annoyance M ± sd (N)**				***p* (Mann-Whitney *U*-Test)**
**Gender**	**Male**		**Female**		
	52.49 ± 12.12	(156)	52.22 ± 11.81	(152)	0.824
**Tinnitus onset**	**Subtle**		**Sudden**		
	52.48 ± 11.58	(114)	52.91 ± 12.39	(130)	0.727
**Symptom reported by patient**	**Yes**		**No**		
Tinnitus onset involving pressure in ears	54.39 ± 11.73	(61)	52.02 ± 11.98	(184)	0.129
Tinnitus onset involving hearing loss	53.43 ± 11.10	(67)	52.52 ± 12.20	(178)	0.433
**Tinnitus onset involving dizziness**	**56.59 ± 12.53**	**(51)**	**51.70 ± 11.49**	**(196)**	**0.007**
**Tinnitus masked trough background noise**	**51.59 ± 11.80**	**(208)**	**54.67 ± 12.06**	**(78)**	**0.050**
Tinnitus gets louder in noise	53.83 ± 12.83	(88)	52.01 ± 11.62	(146)	0.233
Noise sensitivity	53.76 ± 12.42	(176)	53.45 ± 11.11	(107)	0.111
**Physical stress leads to tinnitus change**	**54.54 ± 12.37**	**(109)**	**51.32 ± 11.40**	**(179)**	**0.029**
Emotional stress leads to tinnitus change	52.70 ± 11.97	(211)	52.05 ± 11.93	(77)	0.576
Jaw movement leads to tinnitus change	51.14 ± 12.73	(42)	52.74 ± 11.66	(234)	0.273
Head movement leads to tinnitus change	52.36 ± 11.31	(39)	52.41 ± 11.99	(241)	0.971
Sudden hearing loss in the past	52.57 ± 11.36	(49)	52.32 ± 12.07	(259)	0.739
**Current subjective hearing loss**	**53.66 ± 11.57**	**(196)**	**49.27 ± 12.52**	**(66)**	**0.015**
**Comorbid psychiatric disorder** (ICD-10 Checklist)	**Yes**		**No**		
	**57.65 ± 12.86**	**(63)**	**50.91 ± 11.28**	**(244)**	< **0.001**
**Psychological symptoms (PHQ)**	**High**		**Low**		
**Depressive mood**	**49.31 ± 11.28**	**(112)**	**57.96 ± 11.65**	**(173)**	< **0.001**
**Anxiety**	**50.86 ± 11.50**	**(78)**	**57.62 ± 12.60**	**(207)**	< **0.001**
**Somatic complains**	**49.64 ± 11.68**	**(138)**	**55.97 ± 11.86**	**(147)**	< **0.001**
**Stress**	**51.92 ± 12.19**	**(49)**	**56.18 ± 11.64**	**(237)**	**0.029**

*PHQ, Patient Health Questionnaire (Spitzer et al., 1999); M, arithmetic mean; sd, standard deviation; N, number of patients in each category. Significant values (p < 0.05) in bold*.

**Table 4:** Correlation with tinnitus annoyance measured with Tinnitus Questionnaire at baseline.

**Variable**	**Pearson's *r***	***p***
**Age**	**0.131**	**0.022**
Tinnitus duration	0.109	0.057
**Number of tinnitus sounds**	**0.152**	**0.008**
**Hearing loss in left ear (4 PTA)**	**0.247**	**<0.001**
**Hearing loss in right ear (4 PTA)**	**0.263**	**<0.001**
Tinnitus frequency right	−0.130	0.633
Tinnitus frequency left	−0.101	0.562
**Tinnitus loudness dB right**	**0.631**	**0.021**
Tinnitus loudness dB left	0.125	0.494

*4 PTA, pure tone average for 500 Hz, 1, 2 and 4 kHz. Significant values (p < 0.05) in bold*.

Patients with the following symptoms displayed higher tinnitus annoyance at T0: dizziness at tinnitus onset, tinnitus sound could not be masked with background noise, tinnitus worsening during physical stress (e.g., exercise), subjective hearing loss, comorbid psychiatric diagnosis (ICD-10) as well as acute multiple somatic complaints, depressive mood, anxiety and high stress level according to PHQ (Spitzer et al., 1999). Higher tinnitus annoyance at the first appointment was correlated with higher age and higher hearing loss. While tinnitus loudness (tinnitus matching, dB) in the right ear correlated with tinnitus annoyance at T0 significantly, there was no correlation for the left ear.

### Predictors of treatment success

**Figure 3:**
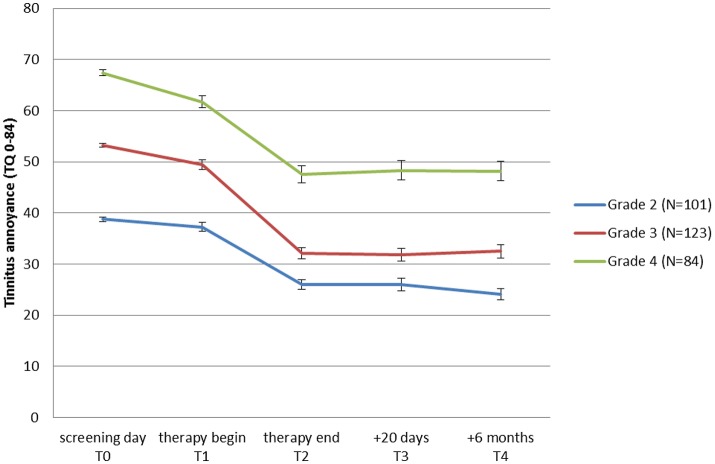
Change of tinnitus annoyance measured with Tinnitus Questionnaire (TQ: Goebel and Hiller, 1998) over time. A higher score represents a higher annoyance. Mean values for patient-groups which started the JITT with moderate (grade 2), severe (grade 3), and very severe (grade 4) tinnitus annoyance are shown. Standard errors of mean are plotted for each point in time and group.

To learn if all tinnitus annoyance subgroups benefit from treatment, patients were classified into 3 groups (moderate, severe, and very severe tinnitus annoyance) depending on their TQ scores at T0, and repeated measures ANOVAs were performed for each subgroup. As can be seen in Table 5 as well as in Figure 3, all three subgroups had a significant overall change in tinnitus annoyance over time (see Data Sheet 1 in the Supplementary Material for individual change in each subgroup). *Post hoc t*-tests with Bonferroni correction showed the following:

**Table 5:** TQ sum scores over time and results of repeated measures analysis of variance (ANOVA) as well as *post hoc t*-tests.

**Tinnitus annoyance**	**T0 Baseline** ***M* (sd)**	**T1 Therapy begin** ***M* (sd)**	**T2 Therapy end** ***M* (sd)**	**T3 1st follow up (2.5 weeks)** ***M* (sd)**	**T4 2nd follow up (6 months)** ***M* (sd)**	***F***	***df***	p^*^
Moderate (*N* = 101)	38.77 (4.46)	37.24 (9.14)	25.98^a^^,^^b^ (10.06)	25.98^a^^,^^b^ (11.84)	24.13^a^^,^^b^ (11.64)	56.21	4/97	**<0.001**
Severe (*N* = 123)	53.24 (3.83)	49.46^a^ (10.09)	32.07^a^^,^^b^ (12.00)	31.77^a^^,^^b^ (13.37)	32.49^a^^,^^b^ (14.91)	118.96	4/119	**<0.001**
Very severe (*N* = 84)	67.39 (5.29)	61.71^a^ (10.77)	47.52^a^^,^^b^ (15.10)	48.32^a^^,^^b^ (17.05)	48.17^a^^,^^b^ (17.59)	49.45	4/80	**<0.001**

a Significant change (p < 0.05 corrected) to baseline according to paired t-test with Bonferroni correction;

b Significant change (p < 0.05 corrected) to T1 according to paired t-test with Bonferroni correction;

* *significant values (p < 0.05) in bold*.

In the subgroup with *moderate tinnitus annoyance* there was no change in tinnitus annoyance from T0 to T1, but from T1 to T2 tinnitus annoyance reduced significantly by 11 points at TQ, changing the grading of tinnitus annoyance from “moderate” to “mild” and remaining constant at T3 and T4. Cohen's effect size of this change from T0 to T4 is *d* = 1.66.

In the subgroup with *severe tinnitus annoyance* a significant reduction of tinnitus annoyance from T0 to T1 by 4 points in TQ was observed, but grading of the tinnitus annoyance did not change. During treatment the significant change of tinnitus annoyance in TQ (17 points) was observed, shifting tinnitus grading from “severe” to “moderate,” achieving a clinical change and remaining stable at T3 and T4. Cohen's effect size of this change from T0 to T4 is *d* = 1.91.

In the subgroup with *very severe tinnitus annoyance* there was a significant reduction in tinnitus annoyance from T0 to T1 by 7 points in TQ, without the change in grading of the tinnitus annoyance. During treatment a further change of tinnitus annoyance in TQ (14 points) was observed leading to a change in tinnitus grading from “very severe” to “severe” with no further reduction at T3 and T4. Cohen's effect size of this change from T0 to T4 is *d* = 1.47.

To analyze, which factors predict the change of tinnitus annoyance during treatment, we used the difference between T0 and T4 in TQ sum score as dependent variable (Bonate, 2000). After including various personal, tinnitus and strain variables as independent variables in a multiple regression analysis, the model could explain only 12.8% of the variance (R Square = 0.128) if all predictors were used. Because this model explained only so little of the variance despite the large number of predictors used, we refrained from using stepwise procedures. The only significant predictors were “sick leave 6 months before treatment onset” (B = 7.190, SE B = 3.268, ß = 0.148, *p* = 0.016) and “tinnitus annoyance at T0” (B = 0.204, SE B = 0.081, ß = 0.171, *p* = 0.012). Including “early change” as a predictor in second regression analysis allowed to explain 27.4% (R Square = 0.274) of the variance with “early change” being the strongest predictor (B = −0.661, SE B = 0.90, ß = −0.429, *p* < 0.001).

## Discussion and conclusion

For most patients with tinnitus annoyance, the only available therapy option at the moment is a basic ENT examination, because all other guideline recommended therapies are hardly available (at least in Germany). Even though German public health insurance covers the cost for CBT for decompensated chronic tinnitus, the barrier to seek help at a psychotherapist (e.g., public, perceived and self-stigmatizing attitudes to mental illness or difficulty identifying the symptoms of mental illness; for more see Gulliver et al., 2010) is high. This significantly reduces the number of those taking part in CBT. Only patients with very severe tinnitus annoyance and comorbid psychiatric diagnosis receive interdisciplinary stationary treatment covered by the German public health insurance.

A fixed duration for a given intervention and the subsequent decrease in the resources needed for accommodation (overnight rooms, beds, meals, night-shift employees, etc.) would reduce the overall costs and makes them predictable. For this reason we developed a 5-day day care treatment program (JITT), in order to fill the need for a broadly available interdisciplinary treatment for tinnitus. We measured the changes in tinnitus annoyance from an initial consultation (T0) up to 6 months after the end of treatment (T4). To summarize the results briefly, the developed treatment is highly promising in reducing tinnitus annoyance and treatment effects remained stable until at least 6 months after the end of the day care program. This was indexed by the generally high compliance and the overall measure for tinnitus annoyance from the TQ. While inspecting this questionnaire in more detail, it turned out that complaints about emotional and cognitive distress, intrusiveness and disturbed sleep, already improved to some degree after initial consultation (T0), but improved even more in response to the day care treatment (T1–T4). In contrast, complaints about hearing and somatic problems improved only upon treatment. High annoyance was characterized at T0 by several somatic and psychiatric symptoms, but predicting the outcome of treatment proved unsatisfactory. We will discuss each of these aspects below.

### General treatment effects and compliance

The general index for tinnitus annoyance demonstrated a considerable reduction from the first consultation (T0) to the final measurement (T4). There was not only a reduction upon the treatment itself, but already earlier, i.e., between the first appointment (T0) and the start of the daycare treatment (T1) corresponding to 4 points in TQ. We assume that this reduction is the outcome of the extensive diagnostic procedures paired with a first, very brief counseling, reassuring the patient that no severe physical abnormality was detected. This information by itself obviously provided some relief. Support for this assumption is provided by a change in the subscales on emotional and cognitive distress as well as intrusiveness and sleep disturbance, but not in the scales addressing hearing and somatic problems. Similarly, waiting for the treatment and certainty of “getting help soon” could have induced this reduction (T0–T1). A meta-analysis of 11 studies included 314 individuals with tinnitus distress that were randomly allocated to a waiting phase lasting 6–12 weeks (Hesser et al., 2011a). The patients revealed a mean decrease in symptom severity between 3 and 8% (Hedges' *g* = 0.17). Thus, already in response to a waiting period tinnitus patients improve slightly on psychometrically robust tinnitus-specific measures.

In our study, the effect size of the overall change before the start of the day care treatment is considered as small. Consequently, at the beginning of treatment the mean tinnitus annoyance was on average still severe. This means that broad and interdisciplinary (ENT doctor, psychologist and audiologist) diagnostics and counseling leads to a significant reduction of tinnitus annoyance, but it does not lead to patients reaching the non-severe range. Perhaps this is why many patients reported during treatment that they were already somewhat relieved after the primary consultation in our clinic or elsewhere, but that they did not know how to cope better with tinnitus in the future.

The change in tinnitus annoyance from the beginning (T1) to the end of treatment (T2) is in comparison to the earlier effect considerable, reaching a large effect size with a change in the TQ sum score of further 15 points (in total 18 points); i.e., the mean tinnitus annoyance at treatment end is in the moderate range. This clinically relevant improvement from a decompensated, clinically severe state to a compensated, moderate state remained stable at follow-ups 20 days as well as 6 months later. In contrast to the different responsiveness of subscales for the early change, all TQ subscales reduced significantly upon treatment. Currently, we have no evidence on which of the modules proved more or less successful, but patients considered all modules important.

In general, reported TQ changes in response to different therapeutic approaches differ widely: between 5.2 points (Rief et al., 2005), 7.8 points (Goebel, 1995), 13 points (Haerkötter, 2001), 18.6 points (Weise, 2008), and up to 23.2 points (Weise et al., 2007). Most often the changes at follow-up are smaller compared to the end of treatment, but still significantly larger compared to the onset of treatment (Jakes et al., 1992). As such, JITT seems highly promising, but we also would like to point out several difficulties in directly comparing the different approaches.

The efficacy of most tinnitus management interventions recommended for clinical practice remains to be demonstrated. Currently, only few studies allow making informed conclusions. The efficacy of therapist-delivered CBT appears to be reasonably established (e.g., Hoare et al., 2011). A multidisciplinary CBT-based approach, in which professionals in audiology and psychology share treatment goals aimed at coping with tinnitus through education and counseling, is likely to optimize the benefit for patients (Cima et al., 2012). Thus, multidisciplinary approach was recommended for some time (Henry and Wilson, 1996; El Refaie et al., 2004; Andersson et al., 2005; Henry et al., 2005; Cima et al., 2009; Hoare et al., 2011).

Nevertheless, there are only few researchers reporting the effects of multidisciplinary treatment, often with the limitation that only inpatients of a specialized hospital were examined (Goebel, 1998, 2008; Hiller and Goebel, 1999; Goebel et al., 2006; Schaaf et al., 2017). These patients are known to be generally more severely impaired and suffer from more psychological complaints than the average patients of ENT practitioners or audiological outpatient departments (Hiller and Goebel, 1999). Therefore, results obtained from inpatient treatment do not seem representative of an outpatient population.

Mazurek et al. (2005) described a 7-day day care interdisciplinary tinnitus-treatment and evaluated it on 46 outpatients. Tinnitus annoyance was reduced significantly from 33.8 points to 27.8 points after 7 days and continued to attenuate to 25.2 points 6 months after treatment. A significant reduction in TQ was observed up to 3 years after treatment (Seydel et al., 2015).

In a large randomized clinical trial, a multidisciplinary stepped care approach involving counseling and elements of CBT and tinnitus retraining therapy (TRT) demonstrated a significant reduction in tinnitus severity (from 49.39 points in TQ to 36.47 points in TQ after 8 months) and tinnitus impairment, as well as improvement of health-related quality of life as compared to usual care (Cima et al., 2012).

Even though the optimal exposure-response relation between number of hours in treatment and outcome remains unknown (Andersson, 2002), the burden for patients and clinicians, as well as the cost-benefit ratio are important (Hoare et al., 2011). JITT lasted 5 days with 2 follow-ups, while other authors report up to 2 years of contact with patients (e.g., Von Wedel and von Wedel, 2000). The very low dropout rate in our study (< 1%) suggests that tinnitus patients were generally satisfied with the treatment and that 5 days of treatment seemed to be a reasonable amount of time patients were willing to invest. Such a low dropout rate is remarkable given the much higher rates reported in a review of CBT for tinnitus patients with dropouts ranging between 5 and 22% (Martines-Devesa et al., 2010). These rates were even higher when CBT was delivered via internet (51% in Andersson et al., 2002; 75% in Abbott et al., 2009), even though there was no difference in the reduction of tinnitus annoyance between internet-delivered or therapist-delivered CBT (Kaldo et al., 2008).

### Predictors of tinnitus annoyance

To describe the heterogeneity of tinnitus patients and in the search for factors related to tinnitus annoyance at T0, we found that patients with the following symptoms displayed higher tinnitus annoyance at the beginning of treatment: dizziness at tinnitus onset, tinnitus sound could not be masked with background noise, tinnitus worsening during physical stress (e.g., exercise), subjective hearing loss, comorbid psychiatric diagnosis (ICD-10) as well as acute multiple somatic complaints, depressive mood, anxiety and high stress level according to PHQ (Spitzer et al., 1999). Additionally, higher tinnitus annoyance at the first appointment was correlated with higher age and greater hearing loss and tinnitus loudness (only for the right ear). This relation is supported by a series of studies that we will briefly review below.

The association between hearing loss and tinnitus corroborates earlier research and is a long standing finding. Prevalence of hearing loss increases with age (Davis, 1995), hearing loss increases the risk for developing tinnitus (Hoffman and Reed, 2004), and on a population level there is a linear increase in tinnitus annoyance with increasing age (Davis and El Rafaie, 2000; Andersson et al., 2005). Studies indicate that 70–80% of tinnitus patients have significant hearing difficulties (Henry et al., 2005; Hesse and Schaaf, 2015). Other studies also reported the positive link between higher hearing loss and higher tinnitus distress (e.g., Goebel and Hiller, 1999; Davis and El Rafaie, 2000; Stobik et al., 2003). For example, Savastano (2008) investigated 520 persons suffering from tinnitus and compared tinnitus patients with and without hearing loss. The author found that subjective discomfort is higher in the presence of hearing loss than in the case of normal hearing (according to Bureau International D'Audiophonologie pure tone average for 500 Hz, 1, 2, and 4 kHz < 20 dB). Among subjects with normal hearing, the level of disturbance was mostly in the moderate range, whereas among subjects with hearing loss, the level of disturbance was mostly elevated. Savastano concluded that the presence of hearing loss increases the complaint of tinnitus considerably, even if the hearing deficit is not severe.

Similarly, the correlation of tinnitus annoyance with otological symptoms reported here corroborates earlier research. Goebel and Hiller (2007) found a strong association between otological conditions and the development of high annoyance: subjects with additional hearing loss and dizziness/vertigo reported both higher loudness and higher annoyance. When subjects with high versus low annoyance were compared, the following odds ratios (OR) were found: hearing loss OR = 5.64 and dizziness/vertigo OR = 3.76. Hallam and Stephens (1985) found that tinnitus patients who complained of dizziness also suffered from higher emotional distress. Both Erlandsson et al. (1992) and Langenbach et al. (2005) observed a worsening of mood and tinnitus symptoms when tinnitus was accompanied by vertigo. The latter study also confirms our finding that loudness of the tinnitus perceived in the right ear correlated with higher tinnitus annoyance at the first appointment. Thus, the sound perceived in the right ear has a stronger impact on the associated emotional processes. This mechanism is not well understood and should be investigated in future studies.

The association between high tinnitus annoyance and poor maskability was also reported in several studies. Goebel and Hiller (1999) reported higher tinnitus annoyance when maskability was poor. Maskability of tinnitus at admission to CBT was a predictor of tinnitus-related distress at a 5-year follow-up (Andersson et al., 2001). Stobik et al. (2005) compared patients with low/moderate and severe/very severe tinnitus distress and also found that patients with severe/very severe tinnitus reported greater difficulty to mask their tinnitus with background sounds.

The relationship between tinnitus and emotional distress or psychiatric problems has long been recognized and is well documented, at least in the help-seeking group (Harrop-Griffiths et al., 1987; Dobie et al., 1992; Andersson, 2002). Sixty-three to seventy-seven percent of tinnitus inpatients have at least one psychiatric diagnosis (mostly mood or anxiety disorder; Kaldo, 2008). The prevalence of concurrent depression or mood disorders ranges between 39 and 60%, whereas the lifetime prevalence amounts to 62–78% (Andersson, 2002). Other psychological causes of distress associated with tinnitus include anxiety, depression, irritability, anger, and insomnia (Wilson et al., 1991). Approximately half of the patients with tinnitus without severe hearing impairment also suffer from psychiatric disorders, the most frequent being anxiety disorders and mood disorders (Zöger et al., 2001). Belli et al. (2008) applied the Structured Clinical Interview for DSM-III-R at 90 acute tinnitus patients and found that 24.4% had at least one axis-I psychiatric diagnosis. The most prevalent disorders were anxiety, somatoform and mood disorders. Only 6% of controls without tinnitus had at least one axis-I psychiatric diagnosis. Depression, sleep disorders and difficulties in concentration were significant predictors of tinnitus annoyance (Scott et al., 1990). Erlandsson and Hallberg (2000) investigated in 122 tinnitus inpatients which factors predict quality of life, and found that impaired concentration, feeling depressed and perceived negative attitudes were the most significant predictors and explained most of the variance in quality of life. In the study from Langenbach et al. (2005) on acute tinnitus, insomnia attributed to tinnitus was the best predictor and accounted for 34% of the variance of tinnitus distress 6 months after tinnitus onset. Anxiety in the acute phase accounted for 30% of variance of tinnitus distress, while life satisfaction and somatic complaints accounted together for 41% of the variance.

We conclude that our results of predicting tinnitus annoyance corroborate previous results. Taking Jastreboff's neurophysiological model of tinnitus into account, the negative impact of these symptoms on developing tinnitus annoyance is quite obvious. As the frequency of the individually perceived tinnitus is very likely to be in the range with the highest hearing loss (Henry et al., 1999; Noreña et al., 2002), the masking of tinnitus with background sounds is, in the case of hearing loss, not possible any more. Habituation inhibits tolerance to a stimulus because of its unpredictability. This is possibly why the variability of tinnitus during physical stress attracts attention to the phantom sound, making it difficult to habituate, which in turn leads to higher annoyance. If a person experiences tinnitus onset simultaneously with dizziness, the fear evoked by dizziness will be associated with the noise according to the principles of classical conditioning. Whenever tinnitus is perceived as a danger, no habituation can be achieved. This risk certainly gets higher, due to the belief that hearing loss or dizziness is caused by tinnitus, which is something that many of our patients reported. In the same direction, emotional distress or psychiatric problems are generally regarded as factors hindering habituation.

### Predictors of treatment outcome

Inspecting changes in tinnitus annoyance in response to treatment, data from patients with moderate, severe and very severe tinnitus annoyance reached high effect sizes. But if we consider only a clinically significant change, JITT displays the strongest effects in patients with severe tinnitus (grade 3). Although tinnitus annoyance is significantly reduced in patients with very severe tinnitus (grade 4), the 5-day treatment is not sufficient to lead to a clinically significant change in this group. Perhaps this group of patients needs an extended duration of JITT or some other outpatient therapeutic approaches. Another possibility would be intensive inpatient care, which however removes patients from their daily routine. Also other researchers found that patients with high tinnitus annoyance at baseline were more often non-responders (Rübler, 1997; Frenzel, 1998) in outpatient setting.

Demographic, tinnitus and strain variables explained only 12.8% of the variance of the change in tinnitus annoyance from T0 to T4. The only predictors for reduction of tinnitus-related distress were “sick leave 6 months before the treatment onset” and “tinnitus annoyance at T0.” Patients who were on sick leave before the treatment or with high tinnitus annoyance at T0 showed less improvement in tinnitus annoyance from treatment begin to the final follow-up. We did not inquire further why the patients were on sick leave, but they most likely experienced somatic/psychological symptoms to such an extent that they were unable to continue with their daily activities. The 5-day treatment was perhaps too short for this subgroup. It is yet unclear if they would benefit from a longer treatment duration or a combination of treatments as suggested below. Including “early change” as a predictor allowed to explain 27.4% of the variance, i.e., considerably more. Early change has generally proven to be a strong predictor in psychotherapy and CBT in particular (Schibbye et al., 2014). We did not include this variable in the first regression analysis, because it already requires knowledge about the responsiveness of a patient which was not given at T0. Nevertheless, it constitutes an early and easy calculable indicator who will respond to treatment and who is more resilient. Providing knowledge about early change to therapists could result in more effective treatment (Lambert and Ogles, 2003). Measuring early change seems well constituted for psychiatric disorders (Schibbye et al., 2014; Koffmann, 2017 for recent references), but it is less known in tinnitus research. We propose to integrate such measures in clinical settings for tinnitus treatment. As examples, the Clinical Outcomes in Routine Evaluation questionnaire (CORE, Evans et al., 2000) and the Outcome Questionnaire-45 (OQ-45; Lambert et al., 2004) in combination with the TQ as a disorder specific measure provide validated tools.

Even though the low predictability for treatment success is unsatisfactory, previous studies similarly failed to predict therapy outcome. Rief et al. (2005) found that age and illness duration had only marginal associations with treatment success. Baseline scores of tinnitus annoyance (TQ), gender of the patient or comorbidity with mental disorders were not significant predictors of outpatient psychological treatment. Neither duration of tinnitus nor the level of sleep disturbance, comorbid psychopathology, hearing problems, or experienced stress level affected the outcome of outpatient treatment (Kröner-Herwig et al., 2006). On the other hand, patients with high tinnitus annoyance and comorbid psychopathology at baseline were more often non-responders (Rübler, 1997; Frenzel, 1998). Goebel (2008) conducted a 15-year follow-up after inpatient tinnitus therapy on 271 tinnitus patients. Noise-induced tinnitus, gender and comorbid psychopathology explained 7.6% of the variance in tinnitus annoyance. Male tinnitus sufferers as well as patients with noise-induced tinnitus and high psychopathology reported higher tinnitus annoyance 15 years after the treatment.

Consequently, it appears that there is only little agreement on what can predict treatment outcome, but in no study was predictability good. The complexity of these processes was again stressed by Caffier et al. (2006). In their study, severely affected tinnitus sufferers showed clear improvements in TQ scores without any age-specific differences. In comparison, the groups of younger and older patients with mild tinnitus severity showed higher reductions in TQ scores in comparison to middle-aged patients between 46 and 56 years. Regarding preexisting tinnitus duration, patients with mild tinnitus annoyance demonstrated a particularly strong reduction of annoyance when the tinnitus lasted for less than 1 year. In contrast, in severely affected tinnitus sufferers, preexisting tinnitus duration did not seem to play a role for treatment success.

These results indicate that in order to predict treatment success by patient characteristics, we have to make subgroups and investigate which combinations of subgroup characteristics lead to better/poorer treatment success. Therefore, the next milestone in tinnitus research should be to update large data registries, into which standardized variables can be entered by independent tinnitus researchers. A tinnitus database has already been established by the Tinnitus Research Initiative (http://www.tinnitusresearch.org/index.php/for-researchers/tinnitus-database) and will be improved and enlarged by the TINNET European research network funded by the COST program (http://tinnet.tinnitusresearch.net/). Such a central database will enable the specification of subgroups of tinnitus patients worldwide, making it more possible to develop individually tailored treatments for tinnitus patients.

## Limitations

Due to the lack of a control group receiving a different treatment, we cannot indicate how effective the present approach is compared to other possible treatments or, in the worst case, if it is due to a placebo effect or the mere passing of time. The interdisciplinary treatment comprised several modules, but whether one of them or a specific combination contributed to the reported effects remains to be tested. Future studies might adopt a dismantling approach, leaving out potentially redundant treatment components. Furthermore, cost-effectiveness studies and equivalence trials should be performed.

Even though the results of this study speak in favor of JITT, it must be noted that 35% of the tinnitus patients seen at the ENT department did not accept “habituation to tinnitus” as an objective of the intervention. Therefore, specific treatment approaches adapted to such patients should also be developed. We propose a combination of two therapeutic methods, one addressing tinnitus distress and the other the symptom itself. As an example for the latter, some evidence was presented that tailor-made notched music training reduces tinnitus loudness (Stein et al., 2016). Neurophysiological models (Jastreboff et al., 1996) suggest the proposed combination as a valuable approach that would also satisfy the needs of patients. As an alternative, there are also non-invasive stimulation methods that seem to ameliorate tinnitus symptoms (for a recent case study see Richter et al., 2017).

## Conclusion

Our interdisciplinary day care tinnitus treatment represents a treatment for patients with chronic tinnitus that reduces tinnitus annoyance. After initial interdisciplinary diagnostic procedures and a first brief tinnitus-specific counseling, a small reduction in tinnitus annoyance was found. A clinically relevant change in tinnitus annoyance was observed between the beginning and the end of treatment and remained stable at least for 6 months.

The best treatment outcome was reached by patients with moderate and severe tinnitus. The improvement in tinnitus annoyance in patients with sick leave within 6 months before treatment onset or with very severe tinnitus annoyance was smaller than for the rest of the investigated population.

Given the high heterogeneity of tinnitus, we predict that the development of adapted JITT to individual needs will be challenging. Additional measurements of neurophysiological correlates might help in understanding which aspects of the symptomatology and the underlying neural network undergo changes in response to treatment and which do not.

## Author contributions

DI: Substantial contributions to the conception and design of the work; the acquisition, analysis and interpretation of data for the work; drafting the work and revising it critically for important intellectual content. Wrote the manuscript. CD: Contribution to the analysis and interpretation of data; drafting the work and revising it critically for important intellectual content. Wrote the manuscript. GV, BM and DR: Substantial contributions to the acquisition and interpretation of data for the work; revising the work critically for important intellectual content. US: Substantial contributions to the conception of the work; revising the work critically for important intellectual content. OG: Substantial contributions to the conception and design of the work; the acquisition, analysis and interpretation of data for the work; revising the work critically for important intellectual content. All authors gave their final approval of the version to be published and agree to be accountable for all aspects of the work in ensuring that questions related to the accuracy or integrity of any part of the work are appropriately investigated and resolved.

### Conflict of interest statement

DI received funding from ISMA GmbH (founder of Terzo hearing therapy) for lecturing their associates about tinnitus. ISMA GmbH had no influence on the presented study. The other authors declare that the research was conducted in the absence of any commercial or financial relationships that could be construed as a potential conflict of interest.
