# Supplementary material for: Results of an Interdisciplinary Day Care Approach for Chronic Tinnitus Treatment: A Prospective Study Introducing the Jena Interdisciplinary Treatment for Tinnitus
Source: Front Aging Neurosci. 2017 Jun 16;9:192. doi: 10.3389/fnagi.2017.00192 (PMC5472663; doi:10.3389/fnagi.2017.00192)
Supplement: Supplementary file 1 [file DataSheet1.pdf]

Results of an Interdisciplinary Day Care Approach for Chronic Tinnitus Treatment: A Prospective Study Introducing the Jena Interdisciplinary Treatment for Tinnitus

Daniela Ivansic*, Christian Dobel, Gerd Fabian Volk, Daniel Reinhardt, Boris Müller, Ulrich Christian Smolenski and Orlando Guntinas-Lichius

***Correspondence:** Daniela Ivansic: Daniela.Ivansic@med.uni-jena.de

Supplementary Figures

Suppl. Figure 1: Individual change of tinnitus annoyance measured with Tinnitus Questionnaire (TQ: Goebel and Hiller, 1998) over time. A higher score represents a higher annoyance.

a) Tinnitus-Patients which started the JITT with moderate (grade 2) tinnitus annoyance.
b) Tinnitus-Patients which started the JITT with severe (grade 3) tinnitus annoyance.
c) Tinnitus-Patients which started the JITT with very severe (grade 4) tinnitus annoyance.

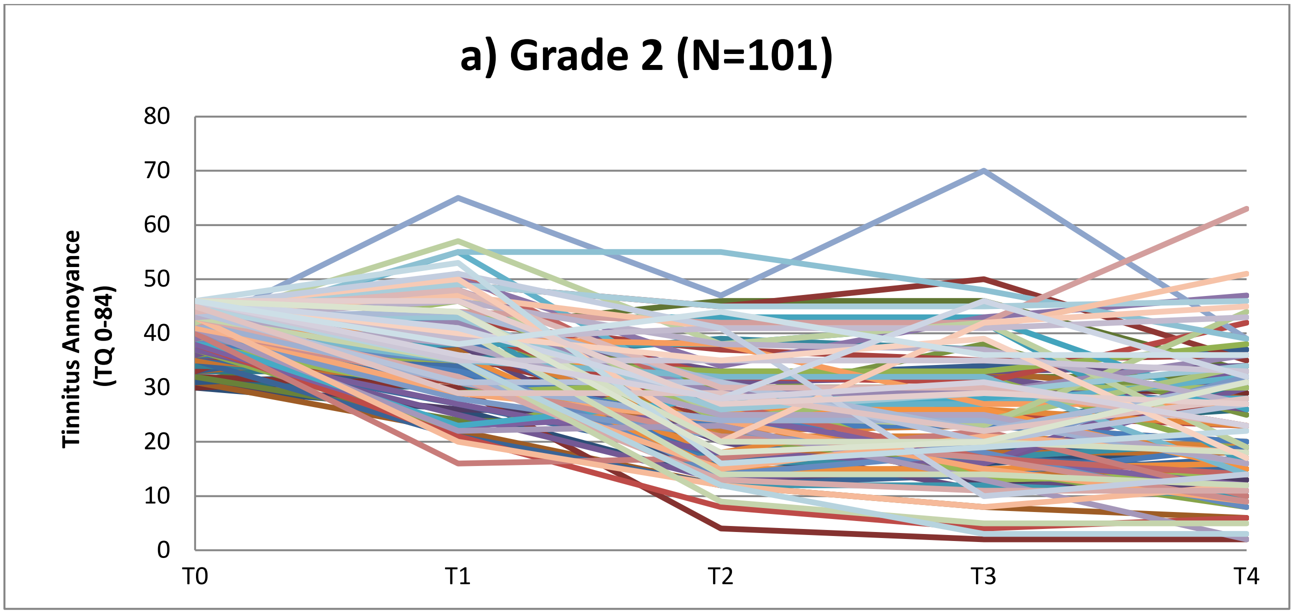

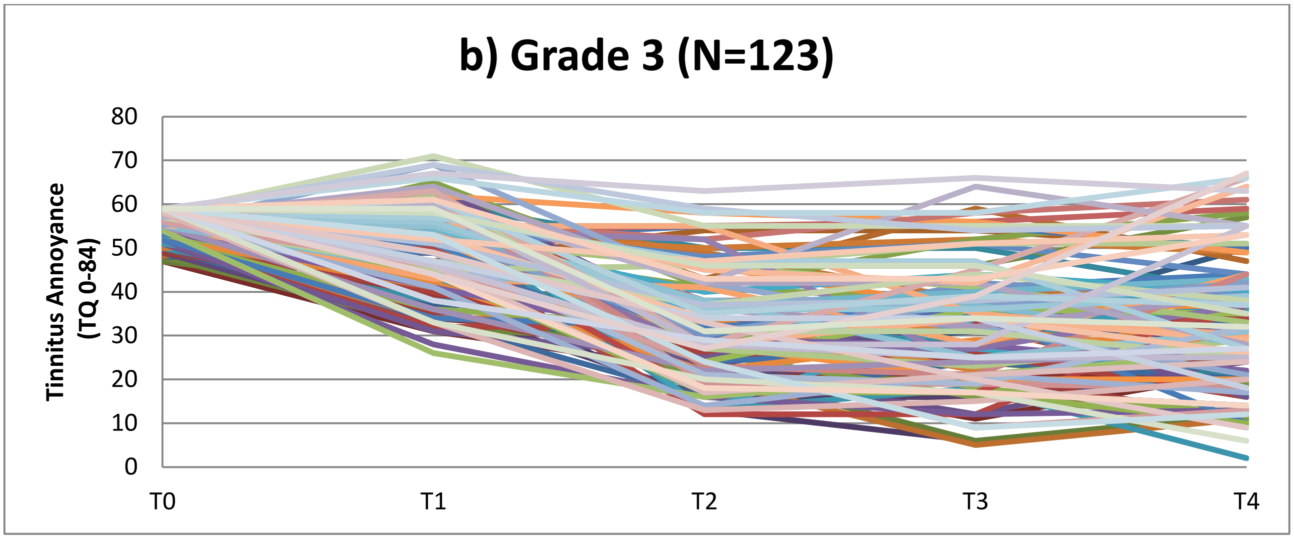
b) Grade 3 (N=123)
Tinnitus Annoyance (TQ 0-84)
80
70
60
50
40
30
20
10
0
T0
T1
T2
T3
T4

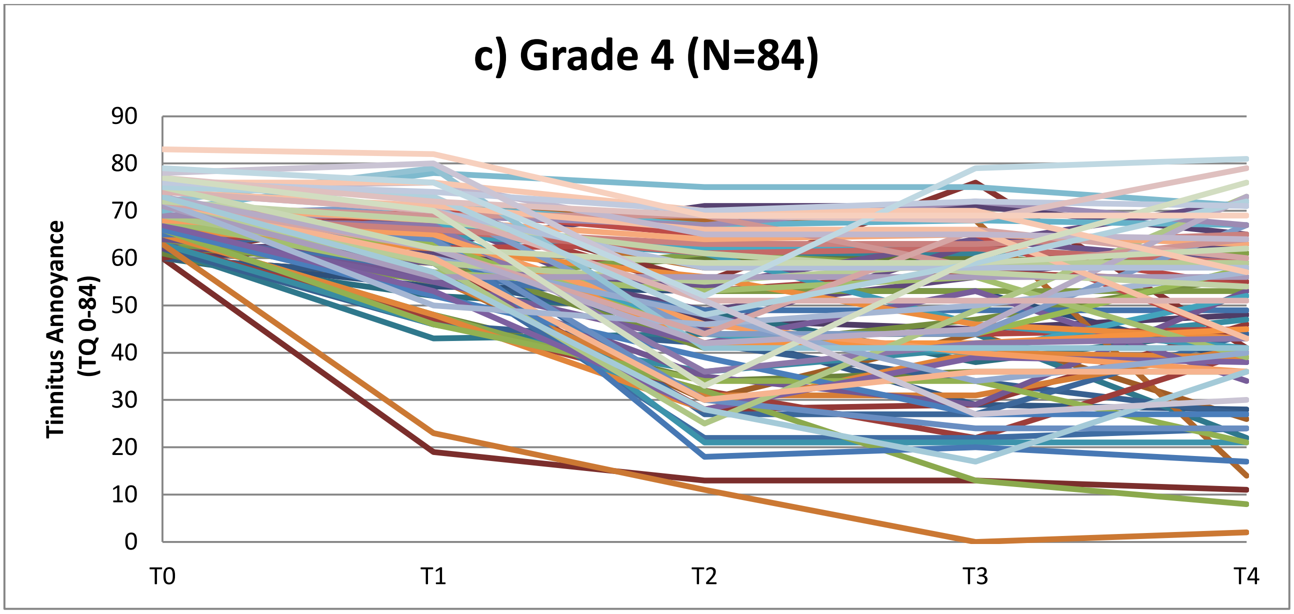
c) Grade 4 (N=84)
Tinnitus Annoyance (TQ 0-84)
90
80
70
60
50
40
30
20
10
0
T0
T1
T2
T3
T4
